# Women and healthcare providers’ perceptions of a midwife-led unit in a Swiss university hospital: a qualitative study

**DOI:** 10.1186/s12884-015-0477-4

**Published:** 2015-03-11

**Authors:** Françoise Maillefer, Claire de Labrusse, Laura Cardia-Vonèche, Patrick Hohlfeld, Beat Stoll

**Affiliations:** University Hospital of the Canton of Vaud (CHUV), 1011 Lausanne, Switzerland; University of Applied Sciences in Western Switzerland (HES-SO, HESAV), 1011 Lausanne, Switzerland; Association Actions en Santé Publique, 1200 Geneva, Switzerland; Maternity Hospital, University Hospital of the Canton of Vaud (CHUV), 1011 Lausanne, Switzerland; Institute of Global Health, University of Geneva, 1202 Geneva, Switzerland

**Keywords:** Midwife-led unit, Maternity services care model, Midwifery, Continuity of care, Physiological childbirth, Qualitative research

## Abstract

**Background:**

The development of medical-led care in obstetrics over the past decades has contributed to improving outcomes for both mother and child. Although efficiency has improved in complex situations, unnecessary interventions are still practiced in low-risk pregnancies, contrary to international recommendations. A shift to a less interventionist model of care has encouraged many countries to review their policies on maternal health care and develop models such as the “midwife-led unit” (MLU) where the midwife plays a predominant role with a minimum of routine intervention. Existing research has provided convincing evidence that MLUs lead to better maternal and neonatal outcomes when compared to traditional models. They not only improve the level of satisfaction amongst women, but are also associated with reduced healthcare costs. This study aimed to explore the perceptions of women and healthcare providers regarding the creation of an MLU in a Swiss university hospital.

**Methods:**

A descriptive research study using qualitative methods was conducted among pregnant women and new mothers in a Swiss maternity unit, including also midwives and medical staff. Data collection was carried out through one-to-one interviews, focus groups, and telephone interviews (n = 63). After transcription, thematic analysis was performed.

**Results:**

The triangulation of perceptions of women and healthcare providers indicated support for the implementation of an MLU to promote physiological delivery. Most women welcomed the idea of an MLU, in particular how it could help in offering continuity of care. Healthcare providers were optimistic about the implementation of an MLU and recognised the need for some women to have access to a less interventionist approach. From the women’s perspective, barriers concerned the lack of awareness of midwives’ full scope of practice, while barriers for midwives and obstetricians were related to the challenge to develop a good interprofessional collaboration.

**Conclusion:**

Alternative models to provide maternity care for low-risk women have been developed and evaluated widely in several countries outside Switzerland. This study showed that women and healthcare providers were favourable towards the development of a new care model, while taking into account the specific expectations and barriers raised by participants.

## Background

In industrialised countries, the hospital has become a privileged place where women give birth [1]. The obstetrician’s involvement and responsibility in normal childbirth has become predominant and, consequently, the number of medical interventions has increased without necessarily being beneficial, particularly among women with low-risk pregnancies [2]. In 1997, the World Health Organization (WHO) issued guidelines regarding care in normal birth and recommended restricting the use of interventions such as the artificial rupture of membranes, use of oxytocics, the number of vaginal examinations during labour, and electronic fetal monitoring [3]. Despite recommendations supported by the United Kingdom (UK) National Institute for Health and Clinical Excellence (NICE), the Royal Australian and New Zealand Colleges of Gynaecologists and Obstetricians (RANZCOG), the New Zealand College of Midwives (NZCOM) and the Royal College of Obstetricians and Gynaecologists (RCOG), the continuous monitoring of women with low-risk pregnancies is still widely performed [4-7]. In parallel, an increase in medical-led care in maternity services has also continued. Therefore, the positions of recognised professional bodies have not been sufficient to reduce the gap between current obstetric practices and existing evidence-based data [8].

While maternal and neonatal mortality rates during childbirth have fallen in developed countries over the past 60 years for either medical or social reasons, the medical supervision of labour and childbirth has long exceeded its efficiency [9,10]. The recent increase in caesarean sections is not linked to health benefits. Instead, it contributes to a rise in the rate of mortality/morbidity [8,11,12] and, in the short term, may affect the relationship between mother and child [13,14]. Furthermore, the mother may encounter emotional sequelae [15]. New challenges for health professionals include avoiding the unnecessary medical care of physiological pregnancies and childbirth [16]. To help overcome this issue, many countries are implementing alternative models of care that improve outcomes for both mother and child, seek to avoid unnecessary medical interventions, and promote normalcy during pregnancy and childbirth [17]. In 2004, the UK National Service Framework for Children, Young People and Maternity Services stated that women should be able to: “choose the most appropriate place to give birth from a range of local options including ....delivery in midwives led units” [18]. In the UK, the proposal outlined in the document “Making it Better for Mother and Baby” page 27 [19] states that women have the choice to either deliver at home, in a local facility under the care of a midwife, or by a local maternity care team. At present, approximately 4% of women in the UK give birth outside hospital facilities, including midwife-led units (MLU). Many countries have thus reviewed their maternal health policies, adjusted their protocols based on proven scientific data, and developed new care models, such as the MLU [19].

An MLU can be defined as giving an emphasis to normality of birth for women who present with a low-risk pregnancy, with midwives as the main healthcare professional [20]. Midwives are trained to observe, identify, and encourage the physiological process of pregnancy and childbirth, and be attentive to any abnormalities [21,22]. The MLU aims to provide care to minimise unnecessary intervention in labour and to involve the woman and her partner(s) in decision making. Active labour, mobilising, upright positions, and eating and drinking are encouraged and will help women to labour successfully. Providing women with the option to have more than one birth companion for support may also minimise stress [20]. The focal point of care delivered in the MLU is to humanize childbirth, as well as promoting self-determination in the woman. The underlying philosophy relies not only on the normality of labour, but also on the continuity of care, with the midwife as the carer who accompanies and monitors the pregnancy and childbirth [23,24].

Switzerland is not excluded from the growing trend of medical interventions. The number of caesarean sections performed in Switzerland is 10% higher than in other European countries, such as France, Germany, Norway and Sweden, which has not contributed to improving the health of either the mother or child [1]. Swiss healthcare presents the specificity to be provided through a compulsory health insurance coverage system [25]. Through their health policies, women have the right to choose the place and the healthcare provider (midwife or obstetrician) for their seven antenatal care appointments [26]. Women with low-risk pregnancies in Switzerland are therefore presented with a diversity of options for antenatal care and place of delivery. Eighteen birth centres are offered throughout the country and approximately 4.3% of births were outside the hospital setting in 2012, which includes MLUs [27,28]. In comparison, the Netherlands have 26 birth centres with 11.4% of births occurring in them [1]. Despite the availability of different options for maternity services, alternative models to medical-led care are not as widely used in Switzerland as it is in other countries such as the Netherlands or UK [1]. Hospitals strive to improve quality services and meet patients’ needs. With 97.5% of births occurring in hospital settings, this mission is even more relevant [29].

The University Hospital of the Canton of Vaud (CHUV) is one of five university hospitals in Switzerland. The increasing intervention rate in the maternity unit is representative of the emergence of medical interventions in normal pregnancy care in Switzerland (Table 1). An evaluation of patient satisfaction conducted at the maternity unit between 2005-2006 highlighted several key areas for improvement: a lack of information provision; lack of homogeneity in information received; and a multiplicity of intervening stakeholders [30].

**Table 1:** **Statistics of interventions at the maternity unit of the University Hospital of the Canton of Vaud in 1991 and 2009**

**Interventions**	**1991**	**2009**
Deliveries (number of births)	1822	2511
Caesarean sections	373 (20.5%)	816 (32.5%)
Instrumental deliveries*	184 (10.1%)	193 (7.7%)
Induced labour	350 (19.2%)	686 (27.3%)
Repeat caesarean sections	153 (8.4%)	686 (27.3%)

*Forceps were the only instruments used at the time for instrumental deliveries.

The purpose of the present study is to explore the perceptions of women and healthcare providers (HCPs) related to the future development of an MLU at our institution. We postulated that obtaining women’s views about their expectations in relation to antenatal care, childbirth, and the postnatal period will aid the development of an adequate service model (such as the MLU) to meet their needs. In addition, obtaining the views of HCPs (midwives and obstetricians) will allow a more in-depth understanding of their perceptions regarding the possible development of an MLU”.

## Methods

### Setting

The study was conducted from 1 March to 30 June 2011 at the CHUV maternity unit, which registers approximately 2700 births per year.

### Design and ethics

To elicit data on the topic of interest, a descriptive research design using qualitative methods (focus groups, one-to-one interviews, and telephone interviews) was selected [31,32]. The study was approved by the ethics committee of the University of Lausanne Faculty Of Medicine in January 2011. Each participant received an information sheet explaining the aims of the study, and a consent form to sign if they agreed to be part of the study.

### Participants

Two groups of participants were recruited: a) women during pregnancy and b) three months after birth; and maternity HCPs, i.e., midwives and consultant obstetricians.

Inclusion criteria for women: >18 years of age with no history of caesarean section in order to be eligible to access the MLU. Additional characteristics: a) women with singleton pregnancies, and b) women who had given birth to one child with no maternal or neonatal pathology at the university maternity unit.

Eligibility criteria for maternity HCPs: all midwives and consultant obstetricians working at the university maternity unit. This accessible population provided a convenience sample.

### Sample size

We aimed to recruit a sample of 40 women (both antenatal and postnatal) to address the research questions. A sample of 10 obstetricians and 20 midwives was considered to be representative of the opinion of the professionals who have expertise in the study topic. This number represents 50% of the potentially available population (the actual available population is unknown, as the numbers of HCPs on holiday, maternity or sick leave could not be identified).

### Recruitment

### Women

During pregnancy, women were invited to participate by the midwives giving the antenatal classes. Approximately 50 women received information about the study; 14 women agreed to take part and participated in two focus groups. Women who had given birth (and who fulfilled the inclusion criteria) were recruited by the postnatal midwives. Forty women were contacted by telephone between one and four weeks after giving birth. Ten women could not be reached or an appointment was not possible. Thirty telephone interviews were conducted.

### HCPs

All midwives of the Department of Obstetrics and Gynaecology and Genetics (DGOG) (n = 50) were contacted by the research team and asked to participate in a focus group as part of the study; 10 midwives agreed to participate. Nine of the 12 available consultant obstetricians of the DGOG were contacted by the research team and agreed to participate in an interview.

### Data collection

Appropriate qualitative research methods were selected according to the participant group. For example, due to time constraints, it was more convenient for obstetricians to be interviewed in a one-to-one interview, while it was more practical for new mothers to do a telephone interview, and more convenient for midwives to participate in a focus group at the beginning or end of a shift. Table 2 presents the qualitative methodology adopted by the participant group.

**Table 2:** **Type of qualitative method related to the type of participant**

**Type of qualitative methods/participants**	**Focus groups**	**One-to-one interview**	**Telephone interview**
Pregnant women (n = 14)	x		
Women during postnatal period (n = 30)			X
Midwives (n = 10)	x		
Obstetricians (n = 9)		x	

The topic guide for focus groups with the women and interviews with the HCPs were developed from the literature and included five questions on the following topics:

- What are your perceptions of the development of an MLU?
- What are the specificities of the MLU?
- What are women’s expectation of the MLU?
- What are your expectations regarding the HCPs working in an MLU?
- What should the MLU guidelines encompass? (for HCPs only)

Focus group discussions and telephone interviews were audio recorded and transcribed verbatim. Field notes taken during one-to-one interviews with the obstetricians were typed up.

### Analysis

Thematic analysis allowed the authors to develop a rich thematic description of this under-researched area. It permitted to identify, analyse and report common themes with a minimum level of interpretation from the researcher [31]. Focus group and telephone interview transcripts were analysed using thematic analysis. The field notes taken during one-to-one interviews with obstetricians were also included in the analysis.

The analytic process included six steps taken from Braun [32]:

1. Preparing the transcript;
2. Generating initial codes by marking words or sentences relevant to the topic of inquiry;
3. From the codes, draft themes were generated in an iterative process;
4. The main categories and sub-categories of the themes were identified;
5. Draft themes were reviewed by the researchers involved in the analysis and defined;
6. Results were written up.

Two researchers collaborated during the analysis phase. Regular discussion of the most recent findings were organised to refine the analysis and reconcile any discrepancies to achieve consensus, thus increasing the comprehensiveness and rigour of the results.

## Results

A total of 63 participants agreed to participate in the study: 14 pregnant women; 30 women who had given birth; 10 midwives; and 9 obstetricians. This section will present the results from interviews and focus groups with women, midwives and obstetricians.

### Focus groups with women

**Figure 1:**
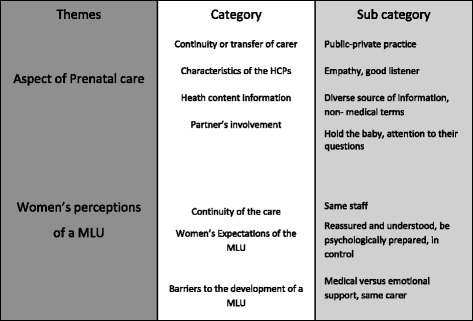
**Examples of subcategories, categories, and themes.** (Inspired by B. Byrne [33]).

Several themes, categories, and sub-categories were identified as summarized in Figure 1 [31,33].

### Aspects of prenatal care

#### Continuity or transfer of carer

Most women who were interviewed agreed that the treatment received in a private practice during pregnancy was better when it was carried out by the same caregiver. Many women found it difficult to be followed by a group of gynaecologists during pregnancy and not to have a designated caregiver who was specifically responsible for them:

*“I was in an office where there were three gynaecologists. Once I was seen by one, then by another, and the next time it was yet another who did the ultrasound. It wasn’t regular and it destabilized me. There was no real continuity. Each time we had to start over to re-establish trust. The laboratory results were not looked at because it was not the same person each time. They basically passed the ball around.” (W16)*

Women often expressed continuity of care as being important, both in private practice and at the CHUV maternity unit. It would allow them to communicate and establish trust in their relationship with the professionals:

*“Each time you have to re-explain the whole pregnancy; give a small biography each time. It is true that it would be much nicer to have the same person. What was important for me was the exchange: to feel at ease and to be able to talk openly about certain things, certain worries. It was mostly about being reassured, being listened to. With this constant change in staff, conversations remain short and we don’t know each other.” (W15)*

Going from a private practice to the CHUV was often reported as being quite easy, although a few women stated it was a difficult transition, especially if they had complications. Two women explained the lack of clear and continuous communication in the context of maternal and fetal complications when having HCPs both in-hospital and outside the hospital:

*“I did not feel able to rely on my doctor who knew me best, but he withdrew from my case when I needed him: when I had no more amniotic fluid. I tried to contact him to discuss it, but he did not call me back. I did not have any other consultations with him. I went to the hospital twice a week.” (W2)*

#### Relations with the HCPs

During pregnancy, women often gave feedback on communication with their HCPs and whether they felt that they have been listened to. Some associated the outcome with having a private insurance, which might, in their eyes, ensure better care.

*“My first gynaecologist was a lout. I know he is an excellent doctor but he has no humanity whatsoever. (….) what is important to me in a gynaecologist are his listening skills, his ability to care for someone – people skills - and less of the cold, medical doctor”. (W1)*

*“When we don’t have private insurance, some gynaecologists send us to the CHUV as quickly as possible and no longer want to hear from us because we are financially uninteresting; that didn’t happen with my gynaecologist.” (W3)*

A welcoming environment, and a professional and human aspect of care is important for women. In general, those who were interviewed spoke positively about the welcoming environment, and the professional and relational competence of the staff, especially the midwives. Women sought empathy and gentleness in their interactions with HCPs. One woman expressed that establishing such a relationship could be difficult, depending on the caregiver.

*“I have given birth twice in the CHUV. On both occasions, I was impressed by the midwife profession because I was in the hands of teams of people whom I found fantastic; women who give birth will remember the people who care for them during those very crucial, and very important moments in their lives; hence, that impressed me.” (W3)*

#### Health content information

After childbirth, the first few days spent in the postpartum unit are important moments in the life of new mothers. During interviews, women expressed the need to have more support in caring for their babies, particularly breastfeeding. Women repeatedly expressed how important it was for them to be reassured by the HCPs and to be able to learn how to care for their newborn babies.

*“As I was leaving, because my baby had regurgitated in the hospital and there were traces of blood, and that they had not explained this further, I asked a nurse about it and I was told it was normal. When a spoonful comes out and it looks as if it is fresh blood, we really panic. Just telling us what might happen would be nice so we do not worry.” (W8)*

Women expected to receive sufficient information and a high level of communication with HCPs. They highlighted the importance of receiving spontaneous explanations using terms and language they understand:

*“In fact they often use medical terms, which we don’t understand, they use words we don’t know at all, they use terms we don’t necessarily understand. They forget that we know nothing about this (laughs).” (W10)*

Many of the women interviewed expressed that they would have liked more information from the HCPs to help them better understand the various phases of pregnancy/child birth:

*“Sometimes, I would have liked to have a little bit more information or at least understand what was happening. I would have also liked to understand what was happening in my body, and also my husband and I would have liked to better understand the situation.” (W12)*

#### Partner’s involvement

Women attributed great importance to the role of their spouses during childbirth. They highlighted how HCPs were attentive to the father and his feelings during childbirth, explaining the procedure to him, and then allowing him to hold the baby as soon as the baby was born:

*“I think that for the first time he felt valued because since it took a little longer, he had the time to stay and we went through the whole labour process together in the hospital. He asked for a few things, to be able to hold the baby on his chest right after she was born, things he had not dared to ask the first time [this was their second child] and which had not been suggested”. (W14)*

The father’s presence in the delivery room was greatly appreciated by the mothers who felt that they were already being well cared for by the HCPs and by their spouses. However, one woman would have liked to have had her husband by her side the night after the birth of their child.

*“It is true that in an ideal world, it would have been great to let my partner sleep there (in the postnatal room), but it was not possible.” (W12)*

### Women’s perceptions of an MLU

#### Continuity of care

Women interviewed were generally optimistic about the creation of an MLU that would ensure continuity of care during their pregnancies and childbirth, including the care given to their baby during their stay. Women expressed that continuous care by the same staff would be both positive and reassuring. An MLU could satisfy the need to normalize childbirth, to render it more natural, and to guarantee emergency care for complications that might arise.

*“What is good in this [the MLU] is that, it will be in the same building as the CHUV and that it could be natural so it’s quite good in case of complications; it is a good idea! It reassures the woman, who at the last minute may worry about something happening to her baby. There can always be a last-minute decision and to be able to decide, for example, to have an epidural is a good idea.” (W17)*

#### Women’s expectations of the MLU

The women clearly expressed their expectations about care at the CHUV maternity unit and how the MLU could satisfy their needs. The women’s expectations of what the MLU should encompass to address their needs are summarised below:

- Be reassured and understood from both a medical and psychological perspective;
- Be assured that if necessary, transfer to a medical unit is available at any time;
- Be psychologically well prepared for childbirth;
- Be able to count on the continuous presence and availability of caregivers;
- Feel more in control about the choice of the analgesic and the positions for childbirth;
- Be able to count on the father’s presence;
- Benefit from a calm environment allowing the family to be together.

#### Barriers to the development of the MLU

Two main barriers to the development of the MLU were highlighted. Some women felt concerned that they would only be cared for by midwives from the beginning of their pregnancy through to childbirth, and feared what might happen if the relationship between them worsened and communication subsequently became difficult. Future mothers reported that they felt safer in the hands of a gynaecologist. Midwives were perceived as being responsible for providing emotional support during pregnancy, accompanying women in childbirth, and providing postpartum care, but not for delivering medical care during pregnancy:

“*Like that, spontaneously*, *I like less the idea to be seen by a midwife during my pregnancy compared to my gynaecologist. However, for childbirth or postpartum, probably yes.” (W14)*

The same woman expressed the need for the MLU to clarify the roles and responsibilities of midwives so that women are aware of and know what to expect from midwives involved in their care:

*“To my knowledge, I feel that we (women) are very gynaecologist-oriented for antenatal care follow-up. I have the impression that people feel that midwives come in at the end. Therefore, we should really be told more about the benefit that this would bring us.” (W14)*

### University hospital midwives

#### Continuity of carer

Midwives working for the DGOG who care for pregnant women felt that continuity of carer leads to both a better recognition of their expert skills and satisfaction of the women. Women need to establish a strong and lasting relationship with their midwife and often express the desire to be accompanied by their midwife during childbirth.

*“With medicalised modern obstetrics, care for women has become fragmented; there is less and less responsibility taken by the woman. A holistic perspective allows for the development of “obstetric intuition” and professional and personal enrichment.” (Focus group midwife, female)*

*“This project corresponds to an increasing demand … especially insofar as continuous care given by the independent midwife during the whole pregnancy is concerned.” (Focus group midwife, female)*

### Meeting the needs of pregnant women and new mothers: an opportunity for health service development

Midwives reported that the MLU model could meet the needs of part of the women in the low-risk category. Even when they appreciated that this population represents a small proportion of the women attending the hospital, they acknowledged that the need for a MLU exists and that this minority will play an important role in the evolution of the MLU. Midwives expressed that the MLU could be an opportunity to meet the needs of women who want personal attention and who request to be personally accompanied by the midwife in a safe environment because the hospital for most women signifies safety. Many midwives felt that the MLU could be the perfect opportunity to introduce a change in maternity service models.

*“This unit would meet the needs of all women with normal pregnancies and who do not want unnecessary medical care. What is important is to be well informed. This unit would be a place where women would be welcomed with their wishes and needs and their competences; women must have confidence in themselves to give birth.” (Focus group midwife, female)*

*“It would allow us to increase and diversify the number of units respecting physiology; women seeking an alternative do not necessarily want to give birth at home or in a birth centre.” (Focus group midwife, female)*

*“It is the right time to instil this change; the population’s needs are evolving. There are more requests for alternative medicine.” (Focus group midwife, female)*

### Professional role and competences

The midwives perceived their current professional role as an opportunity to develop autonomy and to once again become “guardians of physiology”. The competences of those working in the unit and their training were also mentioned:

*“We respect each one’s competences and highlight their independent and autonomous aspects. Interdisciplinary work is reinforced.” (Focus group midwife, female)*

### Change and interprofessional collaboration

The change that the MLU may introduce and the acceptance of the project by obstetricians were seen as risks. Midwives considered what it would be like to collaborate with current obstetricians and paediatricians.

*“Changing habits is challenging: we have to be prepared for resistance and it will take time.” (Focus group midwife, female)*

*“We must reassure the medical staff and integrate them.” (Focus group midwife, female)*

*“We must pay attention to flexibility and communication amongst and between teams.” (Focus group midwife, female)*

Many HCPs questioned how to integrate a unit preoccupied by physiology in a hospital where pathology dominates:

*“The main obstacle is still implementing the model inside the hospital: a physiological island surrounded by a sea of pathology.” (Focus group midwife, female)*

To better prepare women who would like to be accompanied in such a model, the professionals highlighted the importance of building women’s self-confidence and improving the information given and the prenatal preparation.

*“It would be interesting for the woman to be cared continuously by one midwife; also knowing a second midwife or the team, to feel more confident too. Knowing the person who will be present during the birth of the child is a major factor. This is the point in maintaining physiology. Preparation for birth will have to be adapted to the needs of these women (….) the fears of childbirth will have to be discussed in depth, not just rushed over”. (Focus group midwife, female)*

### Obstetricians

From the field notes taken during the one-to-one interviews, the obstetricians favoured the development of an MLU. Nevertheless, they provided some practical suggestions about the process of developing and implementing a MLU in practice. Their comments are grouped into four different themes including facilitators and/or barriers to the development of a MLU: guidelines, interprofessional collaboration, evaluation, and choice of the couple.

### Guidelines

The criteria for transferring patients from the MLU should be precise and strictly respected. Transfers should be anticipated, announced in the delivery room, and fast. Neither the woman nor the midwife should ever consider the transfer as a failure.

### Interprofessional collaboration

All maternity units should work hand in hand and the patients should sense it. Patients should be prepared to meet the medical team, feel confident, and not demonize doctors. Knowing when a pregnancy is abnormal is challenging. The midwife who assumes the role within a MLU must be very competent and experienced. Some obstetricians proposed that a system should be developed and integrated within a MLU where senior midwives were mentors.

At the start of the interview, some obstetricians were concerned that there may not be physiological situations requiring their expertise in standard delivery rooms and feared the consequences of this when training residents. However, by the end of the interview, almost all of the obstetricians interviewed believed that there would be sufficient situations present in standard delivery rooms. Others believed that it could be a training opportunity for new residents, but that it would be important to preserve the couple’s intimacy; it should only be on special request, as an observer, and for a short period of time. They also highlighted that this could lead to organisational challenges.

### Evaluation

All obstetricians interviewed felt that it would be logical to respect physiology and limit the number of interventions practised during normal pregnancies. However, to keep patients in the MLU, they feared that complications might be minimized with a push towards normality in clinical situations that should be transferred to the labour ward. Some proposed to develop an evaluation tool to monitor obstetric outcomes and criteria, as well as the couple’s satisfaction with care.

### Choice of the couple/women

Some obstetricians insisted that being cared for in a MLU must be the choice of the couple/woman. Even in normal physiological situations, some couples/women may still prefer the standard medicalised process.

## Discussion

The purpose of the study was to describe the perceptions of women and HCPs regarding the facilitators and barriers to the development of an MLU. The MLU model has existed for many years and has demonstrated a clear benefit for the health of mother and child through scientific studies [2,34]. As the model does not exist in the canton of Vaud in Western Switzerland, it justified our investigation as to its feasibility in our setting. We sought to explore the opinions of women and various HCPs in the field regarding the development of an MLU, including the acceptability of such a project. The findings of this study could improve the development of a healthcare service, which would better meet the needs of the population [35]. Pregnancy is a period when women are engaged in the healthcare system and can therefore play an important role in assessing the care delivered by maternity services. We included also statements from HCPs to give a more comprehensive picture of the opportunities for the development of a MLU and the barriers to be overcome.

Our findings suggest that women are seeking maternity care that can provide continuity of care and acknowledges the importance of good communication between women and their HCPs [36,37]. Women appreciated and understood the benefits of giving birth in an MLU, which confirms the existing evidence of the effectiveness of this care model [17,20,21]. Women also expressed their need for information that is relevant to them and presented in appropriate and understandable language [38]. The involvement of the women’s partner in the maternity care pathway was highlighted as being important to women interviewed in this study, who referred to their partners as “service users”. The identification of partners as service users was previously highlighted by Sandall et al in 2013 [23]. Therefore, a specific attention should be given to include partners in the maternity care pathway.

Findings from our study revealed that the development of an MLU model is well accepted by pregnant women and first-time mothers. Most women interviewed would have liked to have a follow-up appointment in such a unit. Opportunities for the development of an MLU were seen as a valid option for physiological birth that is not yet provided for some women who are looking for non-interventionist maternity care within the hospital setting.

Midwives working in an MLU are the primary caregivers during antenatal, childbirth, and postnatal care. As a barrier to the development of an MLU, women expressed an ambivalence in their perception that the human aspect of care is important (HCP being a good listener and supportive) and that the midwives they met were particularly welcoming and good communicators. However, women perceived that the midwives’ expertise is mostly related to childbirth and postnatal care, and not so much about antenatal care, which is classically organised with an obstetrician. A recommendation for practice would therefore be to focus on improving women’s knowledge on the scope of midwife activities, in particular antenatal care follow-up and the way in which the skills and competences of the midwife are communicated [39,40].

Women have expressed the importance of continuity of care, reducing the number of interventions, and personalizing the system for those who have chosen this type of care during pregnancy and childbirth [41]. In contrast, the absence of epidural anaesthesia in the MLU was scarcely addressed by study participants. Interestingly, women did not express apprehensions or doubts linked to the unavailability of this pain relief option, thus demonstrating the open-mindedness of women accepting a different model of care for pregnancy and childbirth [42].

Both midwives and obstetricians positively responded to the possible development of an MLU within the maternity department of the CHUV and acknowledged the benefits to women who may use the service in the future. By having the focus of one midwife throughout their pregnancy, women using the MLU may be more satisfied with the healthcare that they receive, their self-confidence may increase, and birth outcomes may improve [23]. Nevertheless, obstetricians expressed their anxiety regarding a service change, development of a unit focusing on physiology within a tertiary unit referral centre for pathologies, and the need for good interprofessional collaboration [43].

HCPs reported that there could be a greater autonomy for midwives in an MLU and this would encourage the physiological birth process. Existing evidence highlights that training midwives who would work in the MLU is crucial to its success [44]. Of note, the responsibility conferred upon midwives within an MLU implies a greater accountability. Currently, this responsibility is shared between obstetricians and midwives, which can be confusing. There is a risk that each HCP will rely on the colleague who is primarily responsible for the patient. Accountability entails a re-appropriation of the role of the midwife as defined by the law [45,46]. This new model provides an alternative to the usual physiological birth in hospital, which guarantees optimal safety conditions due to the proximity of the technical plateau; it also satisfies the couple’s needs. Midwives and obstetricians highlighted also that communication plays an important role in the quality of the exchanges between those involved. HCPs highlighted the need to develop an evaluation tool that will permit to evaluate obstetrical outcomes as well as patient satisfaction.

### Strengths and limitations

To the best of our knowledge, this is the first study reporting descriptive qualitative findings related to an exploration of women and HCPs’ perceptions of the development of an MLU in Switzerland. One of the main strengths is that it includes not only women’s perceptions, but also those of HCPs who would deliver care in this unit. The study results provide precious insight concerning the opportunity to develop a service to respond to women’s needs, including HCPs’ current feelings about the development of an MLU, and will help inform strategic planning at the organisational level. Finally, the data came from a variety of sources (women, midwives and obstetricians) and required several data collection methods (focus groups, one-to-one interviews and telephone interviews) which contributed to the richness of the results, but was time intensive. A study limitation is that the views of migrant women are not represented. Nevertheless, the views of independent midwives caring specifically for migrant women were collected in the same topic and will be presented elsewhere.

## Conclusion

The findings of our study indicate that both women and HCPs have positive views on the development of an MLU within a hospital setting, notably concerning continuity of care. Several barriers were highlighted and range from developing trust in midwives who are not visible today as leaders in physiological antenatal care to interprofessional collaboration.

Proposing a new model for maternity care services within a university hospital ensures safety and scientific legitimacy, not only for women and their families, but also for obstetric professionals. In turn, they will transmit different perspectives than those of fear and risk. In parallel and prior to the creation of an MLU, “re-conceptualizing” childbirth is needed. This is a paradigm shift that goes far beyond the hospital: switching from a medical concept to women-centred care that enhances women’s resources.

The results of this study are encouraging and will inform maternity and hospital administrators hoping to facilitate the development of this model of care. The challenges evoked will need to be evaluated using the recommendations of the HCPs involved in this study. We acknowledge the complexity of the issues related to the development of such a unit and recognise that further studies are needed to evaluate the wide range of opportunities and barriers for the development of an MLU in a hospital setting.
